# Poweromin X Ten, a polyherbal formulation improves male sexual function: *In vivo* and network pharmacology study

**DOI:** 10.12688/f1000research.145889.2

**Published:** 2024-08-28

**Authors:** Sree Lalitha Bojja, Kiran Kumar Kolathur, Bhim Bahadur Chaudhari, Gangadhar Hari, Bharath Harohalli Byregowda, Sai Teja Meka, Esekia Raja Selvan, Sudheer Moorkoth, Nitesh Kumar, Anoop Austin, C. Mallikarjuna Rao

**Affiliations:** 1 Department of Pharmacology, Manipal College of Pharmaceutical Sciences, Manipal Academy of Higher Education, Manipal, Karnataka, 576104, India; 2 Department of Pharmaceutical Biotechnology, Manipal College of Pharmaceutical Sciences, Manipal Academy of Higher Education, Manipal, Karnataka, 576104, India; 3 Department of Pharmaceutical Quality Assurance, Manipal College of Pharmaceutical Sciences, Manipal Academy of Higher Education, Manipal, Karnataka, 576104, India; 4 Research & Development centre, apex laboratories private limited, B-59, SIPCOT Industrial Park, Irugattukottai, Kanchipuram District, Tamil Nadu, 602117, India; 5 Department of Pharmacology & Toxicology, National Institute of Pharmaceutical Education and Research, Hajipur, Bihar, 844102, India

**Keywords:** LC-MS/MS, Network pharmacology, Polypharmacology, Reproductive toxicity, Sexual activity, Dopamine

## Abstract

**Introduction:**

Poweromin X Ten (PXT) is a polyherbal formulation, traditionally used to enhance male sexual function. However, the safety and benefits of PXT have not been scientifically evaluated. Therefore, the present study investigated the toxicity and aphrodisiac potential of PXT in male rats and explored its principal mechanisms of action.

**Methods:**

Male Wistar rats were orally administered PXT (50 or 100 mg/kg) for 28 days, and sexual activity parameters, including latency and frequency of mounting and intromissions, were studied. The reproductive toxicity and spermatogenic potential were also examined. Furthermore, dopamine and serotonin levels in brain regions associated with sexual activity were assessed. Network analysis was used to identify the key bioactive compounds and their core targets involved in their beneficial actions.

**Results:**

Treatment with PXT improved sexual activity in male rats, as evidenced by reduced mounting and intromission latency and a significant increase in mount frequency. Moreover, PXT exhibited spermatogenic potential and did not induce reproductive toxicity. Notably, treatment with 50 mg/kg PXT elevated dopamine levels in median preoptic area and hypothalamus. Pathway analysis indicated that PXT primarily modulated the PI3K-Akt, calcium, and MAPK signalling pathways to enhance male sexual function. Network analysis identified macelignan, β-estradiol, testosterone, and paniculatine as key bioactive components of PXT, which likely act through core targets, such as androgen receptor (AR), Mitogen-activated protein kinase 3 (MAPK3), epidermal growth factor receptor (EGFR), estrogen receptor 1 (ESR1), and vascular endothelial growth factor (VEGF) to facilitate the improvement of male sexual function.

**Conclusion:**

Study results suggest that PXT is a safer alternative with aphrodisiac and spermatogenic potential. These effects are partly attributed to the enhanced dopamine levels in the brain. Furthermore, this study provides insights into the specific signalling pathways and bioactive compounds that underlie the improvements in male sexual function associated with PXT.

## 1. Introduction

Sexual activity is essential for various aspects of physical and psychological health, including cardiovascular ^1^ and immune function. ^2^ The sexual response cycle in men comprises sexual desire, arousal, erection, and sexual activity, which, when negatively affected, can result in sexual dysfunction. ^3^ A variety of factors, such as psychological, neurological and vascular diseases, trauma, surgery, and drugs can lead to sexual dysfunction. ^4^ Globally, 20–30% of adult men reported having at least one major sexual disorder, and its prevalence increases with age. ^5^ With increasing stress and anxiety, unhealthy lifestyles, pollutants, and importantly comorbidities, the prevalence of sexual disorders has rapidly increased. ^4^

As sexual disorders continue to increase at an alarming rate, there lies a huge dearth of effective treatments. Only a few drugs exist to improve sexual behavior, one of them is sildenafil citrate, which has gained popularity for treating erectile dysfunction and also as a libido booster. However, this drug causes a number of limiting side effects such as arrhythmias, headaches, facial flushing, blurred vision, and mental confusion. ^6^ In such instances, traditional medicine offers several plant-based drugs, such as Safed Musli, Ashwagandha, Gokshur, *etc*. which have been safely used as aphrodisiacs for ages. These plants are reported to contain bioactive compounds, such as saponins, flavonoids, phenols, alkaloids, and volatile oils, which might improve sexual behavior and treat sexual disorders. ^7^

Poweromin X Ten (PXT) is a marketed polyherbal formulation used to improve male sexual function in healthy populations. It is composed of extracts from medicinal plants of aphrodisiac value, including *Tribulus terrestris* fruit, ^8^ *Withania somnifera* root, ^9^^,^^10^ *Asparagus racemosus* root, ^11^^,^^12^ *Punica granatum* seed, ^13^^,^^14^ *Myristica fragrans* mace, ^15^ *Chlorophytum arundinaceum* root, ^6^ *Cuculigo orchoides* rhizome, ^16^ *Celastrus paniculatus* seed, *Orchis mascula* root, and Shilajit exudate, ^17^ at a standard ratio. However, the safety and beneficial claims of PXT have not yet been scientifically evaluated. Furthermore, the principal phytoconstituents and mechanisms through which they improve male sexual function remain unknown. Therefore, the current study aimed to investigate the safety and efficacy of the PXT formulation in male Wistar rats. In addition, the mechanisms and key targets of PXT in male sexual function were identified using a network pharmacology approach.

## 2. Methods

### 2.1 Institutional animal ethics approval

The study protocol was approved by the Institutional Animal Ethics Committee (IAEC/KMC/50/2022 dated 26.03.2022), Manipal Academy of Higher Education (MAHE), and the experiments were carried out at the Central Animal Research Facility (CARF), Manipal, Karnataka, India (CPCSEA Reg no. 94/Po/ReBi/S/1999/CPCSEA). Male Wistar rats (180–200 g) bred at the CARF, MAHE, Manipal, were procured for this study. Rats were housed two per cage in polypropylene cages and maintained under standard laboratory conditions, 22 ± 2° C, RH 55-70% with 12 h light and dark cycle as per CCSEA (Committee for Control and Supervision of Experiments on Animal) guidelines. Rats were fed a standard pellet diet and water ad libitum. The study was conducted according to ARRIVE guidelines 2.0, using the ARRIVE Essential 10 checklist for pre-clinical animal studies, and all efforts were made to ameliorate any suffering of animals during the experiments. ^18^

### 2.2 Materials

The Ayurvedic formulation, PXT, was sampled by apex laboratories private limited (Green Milk Concepts), Kancheepuram, Tamil Nadu, India. Sildenafil Citrate (Penegra 50) and thiopental (100-150 mg/kg, i.p.) were procured from K. M. C. Pharmacy, Manipal. PXT was dissolved in 0.25% (w/v) carboxymethyl cellulose and administered orally.

Dopamine hydrochloride (655651-10 mg), serotonin hydrochloride (14927-25 mg) and isoprenaline (PHR2722, 250 mg) were purchased from Sigma-Aldrich (St. Louis, MO). Mass-grade formic acid, acetonitrile, and ammonium acetate were purchased from Merck (Germany). Milli-Q water was used for all reagent preparations.

### 2.3 Preparation of standard extract of PXT

Hydroalcoholic dry powder extracts processed from *W. somnifera root, A. racemosus root, P. granatum seed, M. fragrans mace, C. arundinaceum root, O. mascula root, C. orchoides rhizome, T. terrestris fruit, C. paniculatus seed*, and Shilajit exudate were obtained from Amsar Private Limited, India, and the details of their individual specifications and quality standards are provided in the repository. ^19^ The powders along with pharmaceutical excipients were combined at specified ratios into a filing-coated tablet of 800 ± 25 mg for Code No. HTB020, which was used. The tablets were evaluated for a disintegration time of approximately 30 minutes, water solubility of 40%, and loss on drying of 10%. The HPTLC Chromatogram was screened and standardized for the formulation. The tablets were pulverized and used in this study. Batch number HTB020/014 was used in this study. The HPTLC chromatogram of the methanolic extract of the formulation and the respective individual plants are provided in the extended data. ^19^

### 2.4 Acute toxicity study

A single-dose toxicity study was carried out according to the Organization for Economic Co-operation and Development (OECD) guideline 425. ^20^ The maximum dose chosen for the limit test was 2000 mg/kg. Ten male rats were divided into two groups (n=5 each): vehicle (CMC) and treatment (PXT). One rat was dosed at a given time. Following overnight fasting (only food was withheld), PXT (2000 mg/kg) was administered orally. An equal volume of vehicle was administered to the vehicle group. After treatment, the animals were observed continuously for 14 days for any gross behavioral changes or death. Rats were sacrificed (using high-dose thiopental anaesthesia) and the weights of the brain, heart, liver, spleen, lung, kidney, and testes were noted to calculate the organ indices. Relative organ weight was calculated as follows: (organ weight (g)/body weight of the animal on the day of sacrifice (g)) × 100. ^21^

### 2.5 Sexual behavior studies

*2.5.1 Grouping and treatment schedule*

24 healthy male Wistar rats (age~100 days) were randomly divided into four groups (n=6): vehicle control (0.25% CMC), standard control (Sildenafil Citrate 5 mg/kg), and treatment groups (PXT 50 mg/kg and 100 mg/kg). The resource equation method of sample size calculation was used to calculate the sample size. Simple randomization was performed using Microsoft Excel to allocate the animals to various groups. The maximum recommended human dose of PXT is 1 g/day. ^22^ Sildenafil dose was selected based on previous reports. ^8^ After an initial acclimatization period of 7 days, all rats were orally administered their respective treatments for 8 weeks. During the first 10 days of treatment, the rats were trained twice for sexual activity in the presence of an observer blinded to the treatment allocation. Sexual behavior and reproductive toxicity were assessed on 28 ^th^ and 56 ^th^ day, respectively.

*2.5.2 Screening of female rats*

Young female rats (3-4 months old) were screened for regular estrous cycle. ^23^ Briefly, vaginal rinses were collected after washing the vagina with 10 μl of normal saline using a plastic pipette. Rinses were smeared over a glass slide and observed under 10× and 40× of a light microscope. Three types of cells can be observed: round and nucleated epithelial cells, which indicate the proestrus phase, enucleated irregular cornified epithelial cells, which indicate the estrous phase, and dominant presence of leukocytes, indicating the diestrous phase.

*2.5.3 Sexual behavior testing*

Sexual behavior experiments were conducted from 19:00 h to 21:00 h in a transparent acrylic chamber in a dark room with a red dim light that allowed observation. ^6^^,^^24^ Ninety minutes after treatment, each male rat was introduced into the observation chamber and allowed to habituate for 10 minutes. Following this, one sexually receptive female rat (in the estrous phase) was introduced, and sexual behavior was noted for 30 minutes or until the first ejaculation. Following training, sexual behavior was assessed on the 28th day. The following parameters were recorded.

1. Latency for mounting: The time between the first encounter of a receptive female in the arena and the first mount. Mount is when the male rat mounts the female from the back and clutches her flank with his front feet. This was used as a measure of libido or sexual desire.
2. Latency for intromission: The time between the first encounter of a receptive female in the arena and the first intromission. An intromission occurs when the male achieves vaginal insertion during mounting. This was used as a measure of libido or sexual desire.
3. Mount frequency: The number of mounts to ejaculation (there is no vaginal insertion during a mount). This is a measure of sexual drive.
4. Intromission frequency: The time between the first encounter of a receptive female in the arena and the first vaginal insertion. It is a measure of sexual arousal and erectile function.
5. Ejaculation latency: Time taken from first intromission to ejaculation. This is a measure of copulatory efficiency. ^6^^,^^24^

### 2.6 Reproductive toxicity

Toxicity in the reproductive system was measured after 56 days of PXT administration. Sperm count, sperm motility, sperm morphology, organ indices of the testes and seminal vesicles, and histopathology of the testes were performed to assess signs of reproductive toxicity. ^6^ The cauda epididymis was isolated for sperm analysis. Seminal vesicles and testes were isolated for organ index calculation.

*2.6.1 Sperm motility*

Briefly, the cauda epididymis was placed in a petri dish containing 1 ml of high-glucose DMEM (Himedia, 11965092-20 ml) medium. It was cut at a few sites with a surgical blade to allow sperm diffusion. The tubule part was absorbed in DMEM to avoid contact between spermatozoa and air. Immediately, 50 μl of the sperm suspension was placed on a Neubauer hemocytometer. Fifty spermatozoa were observed in each of the four quadrants for forward motility, flagellar beating, or inertness within 5 minutes. The numbers of motile, wiggling, and non-motile sperm were counted. ^6^

*2.6.2 Sperm morphology*

Ten microliters of sperm suspension along with 10 μl of 0.5% eosin Y dye were placed on a clean slide. The smear was then made and air-dried. A total of 200 spermatozoa were observed for abnormalities under a bright-field microscope. Slides were observed for primary abnormalities such as headless tail, alterations in size and shape of the head, and secondary abnormalities such as a tailless head, kinked tail, and bent neck. ^6^

*2.6.3 Sperm count*

Spermatozoa were allowed to diffuse into the medium for 10 minutes. The sperm suspension was 10-fold diluted, and 50 μl of the diluted suspension was used for counting using an improved Neubauer chamber. The total number of spermatozoa was counted in four quadrants and multiplied by the dilution factor. ^6^

*2.6.4 Histopathology*

Following sacrifice, rat testes were isolated and stored in 10% neutral buffered formalin for further histological assessment. Later, testes were paraffin-embedded and sectioned coronally for hematoxylin and eosin (H&E) staining. The slides were then observed under a light microscope for histological changes.

### 2.7 Brain serotonin and dopamine estimations using LC-MS/MS

*2.7.1 Brain homogenate and sample preparation*

Concentrations of serotonin and dopamine in the brain regions responsible for sexual activity were estimated using LC-MS/MS. ^25^ The median preoptic area and hypothalamus were isolated and frozen immediately at -80°C until further analysis. Briefly, brain samples were weighed and homogenized using Polytron PT3100D (Kinematica, Lucerene, Switzerland) at a concentration of 1g tissue/10 ml of 2% formic acid in water (ice cold) and centrifuged at 15000 rpm, 4°C for 20 minutes. The clear supernatant was stored at -20°C until analysis. For the analysis of samples, into a 1.5 ml conical centrifuge tube, 10 μl of isoprenaline (100 ng/ml), 100 μl of brain homogenate, and 400 μl of cold acetonitrile containing 1% formic acid were taken serially. The contents were vortexed for 30 s and centrifuged for 15 minutes at 12000 rpm maintaining at 4°C, and 5 μl of supernatant was injected into the LC-MS (Supplementary 1.2. section).

*2.7.2 LC-MS/MS analysis*

The analysis was performed using a Dionex Ultimate3000 liquid chromatography coupled with a linear ion-trap LTQ-XL mass spectrometer. The Chromatographic separation was achieved using HPLC column (Synergi™ 4 μm Polar-RP 80 Å 150 × 4.6 mm) operated at 25°C at a flow rate of 0.4 ml/minutes with gradient programming; initially, 85%A for 0.5 minutes, linearly changing to 40% A at 2 minutes, kept constant up to 5 minutes, and brought back to 85%A at 6 minutes and conditioned with a total run time of 10 minutes. The mobile phase contained 0.1% formic acid in water (A) and 0.1% formic acid in methanol (B). The mass spectrometer consisted of a heated electron spray ionization source operated in positive polarity at a source heater temperature of 410°C, sheath gas flow of 50 arb, auxiliary gas flow of 16 arb, and capillary temperature of 350°C in consecutive reaction monitoring mode ( Table 1). The method was linear in the range of 5.0 to 425.0 ng/ml in brain homogenates. Because the analytes were endogenous, pooled brain homogenates from the control group were used for the preparation of calibrators, and a background subtraction approach was used for the calculation of concentrations.

**Table 1.** Mass Spectrometer scan event.

Analyte	Precursor ion (m/z) & MS/MS scan	Collision energy %	
		MS2	MS3
Serotonin	m/z 176.90 → 160.00 → 131.90	72.0%	33.0%
Dopamine	m/z 154.16 → 137.00 → 119.00	53.0%	34.0%
Isoprenaline (Internal Standard)	m/z 212.11 → 194.05 → 152.00	20.0%	30.0%

### 2.8 Network pharmacology

*2.8.1 Collection and target prediction of bioactives*

The phytochemical constituents of PXT components, *W. somnifera* root *, A. racemosus* root *, P. granatum* seed *, M. fragrans* mace *, C. arundinaceum* root *, O. mascula* root *, C. orchoides* rhizome *, T. terrestris* fruit *, C. paniculatus* seed, and shilajit exudate were obtained from Dr. Duke’s Phytochemical and Ethnobotanical databases (USDA) ( https://phytochem.nal.usda.gov/phytochem/search/list) and Indian Medicinal Plants Phytochemistry and Therapeutics (IMPPAT, https://cb.imsc.res.in/imppat/) ^26^ and literature survey. The SMILES of the obtained phytocompounds were retrieved from the PubChem database ( https://pubchem.ncbi.nlm.nih.gov/). The phytocompounds were screened for bioavailability and drug-likeness using the SwissADME database. ^27^ Chemical compounds with bioavailability scores >0.55 and more than two out of rule-of-five (Lipinski, Ghose, Veber, Egan, and Muegge) were selected as bioactive compounds. Then, the Swiss Target Prediction (STP) database ( http://www.swisstargetprediction.ch/), ^28^ PharmMapper ( https://www.lilab-ecust.cn/pharmmapper/), ^29^ and Similarity ensemble approach ( https://sea.bkslab.org/#:~:text=Similarity%20ensemble%20approach%20(SEA),build%20cross%2Dtarget%20similarity%20maps) ^30^ were used to predict the targets of bioactives. Targets with a probability greater than 0.01 were considered for STP, whereas all the targets obtained from PharmMapper and SEA were included in the study.

*2.8.2 Target phishing*

Similarly, genes/targets related to male sexual function (MSF) were screened using the query term” male sexual function’ in GeneCards ( https://www.genecards.org/) ^31^ and NCBI gene list ( https://www.ncbi.nlm.nih.gov/gene/). Only genes with a relevance score of > 10 were considered for GeneCards, whereas all the genes obtained were considered for the NCBI gene list. The relevance score is the score through which the GeneCards database ranks all genes associated with the search term.

*2.8.3 Common targets and construction of the Bioactive-target-MSF network*

The collected bioactive and MSF targets were overlapped to identify potential targets for the improvement of sexual function. To explore the interrelationships between PXT bioactives, target genes, and MSF, a compound-target-MSF was created using Cytoscape 3.9.1 software. ^32^ Using a network analyzer, the degree of the network was assessed to identify the key bioactive components of PXT that are involved in male sexual function.

*2.8.4 Gene Ontology (GO) Function Enrichment and KEGG Pathway Analysis*

The Database for annotation, visualization, and integrated discovery (DAVID) software was used to generate functional pathways using GO and KEGG pathway enrichment analyses ( https://david.ncifcrf.gov/tools.jsp). ^33^ The overlapping targets were subjected to DAVID to perform functional annotation (cellular component, molecular function, and biological process). Statistically significant terms (p≤0.01) were identified, and the top 15 GO enrichment and the top 15 KEGG pathways were selected for further analysis. These pathways may be the functional mechanism of the drug, and a network was constructed using Cytoscape.

*2.8.5 Construction of target protein-protein interaction (PPI) Data*

To identify potential targets of the drug, the overlapping targets were mapped to the STRING database version 11.0 ( https://cn.string-db.org/) to obtain the potential PPI network. ^34^ Targets related to Homo sapiens with a high confidence score > 0.4 were selected. PPI network data were exported to the Cytoscape software. The MCODE cluster analysis plugin was used to identify the prominent subregions of the PPI network. Cluster analysis was performed with a node score cutoff=0.2, K-core=2, and degree of cutoff=2. Furthermore, the Maximal Clique Centrality (“MCC”) algorithm in the “CytoHubba” plug-in was used to identify core targets from the prominent cluster, as it has high prediction accuracy. ^35^

### 2.9 Molecular Docking

To verify the results from the network pharmacology studies, the binding affinity between the core targets and their related bioactive compounds was assessed. Crystal structures of the core targets AKT1 (PDB ID: 2UVM), mitogen-activated protein kinase 3 (MAPK3 PDB ID: 2ZOQ), epidermal growth factor receptor (EGFR PDB ID: 1M17), vascular endothelial growth factor (VEGFA PDB ID:1VPF), androgen receptor (AR PDB ID:1T65), and estrogen receptor 1 (ESR1 PDB ID:2BJ4) were retrieved. PDB format from the RCSB Protein Data Bank. Furthermore, the protein structures were preprocessed and prepared for docking in Schrödinger Maestro. ^36^ A protein preparation wizard was used to preprocess the protein by filling the missing side chains and loops if any were missing in the PDB. The OPLS3e force was used to optimize the H-bonds to generate a low-energy-state protein and remove water molecules. According to the component-target interaction network, the top ten bioactive compounds were selected as ligands for docking. The structures of these compounds were retrieved from literature. sdf format from the PubChem database. Furthermore, using LigPrep available in Schrödinger Maestro, three-dimensional (3D) structures of the selected compounds were generated. ^37^ The SiteMap tool was used to generate a grid for proteins without the co-crystallized ligand. ^38^ A grid was generated around the site with the highest SiteScore, which had the requisite amino acid residues to modify protein activity. Glide’s extra precision (XP) and rigid docking were employed, and the docking score was recorded for each ligand with different core proteins. ^39^ Prime MM-GBSA calculations were performed to estimate the binding energies between the ligand and receptor interactions. ^40^ 2D amino acid interactions of the ligand-protein complexes with better docking scores were noted.

### 2.10 Statistical analysis

GraphPad Prism 8.0 software (SRC00008- https://www.graphpad.com/) was used for statistical analysis. Copyright license for the GraphPad Prism software was obtained. R is an open source alternative software which could be used for data analysis. Data were depicted as mean ± standard error of the mean (SEM). One-way analysis of variance (ANOVA) test followed by the Tukey multiple comparison test was applied. p<0.05 was considered statistically significant.

## 3. Results

### 3.1 Acute toxicity

**Figure 1.**
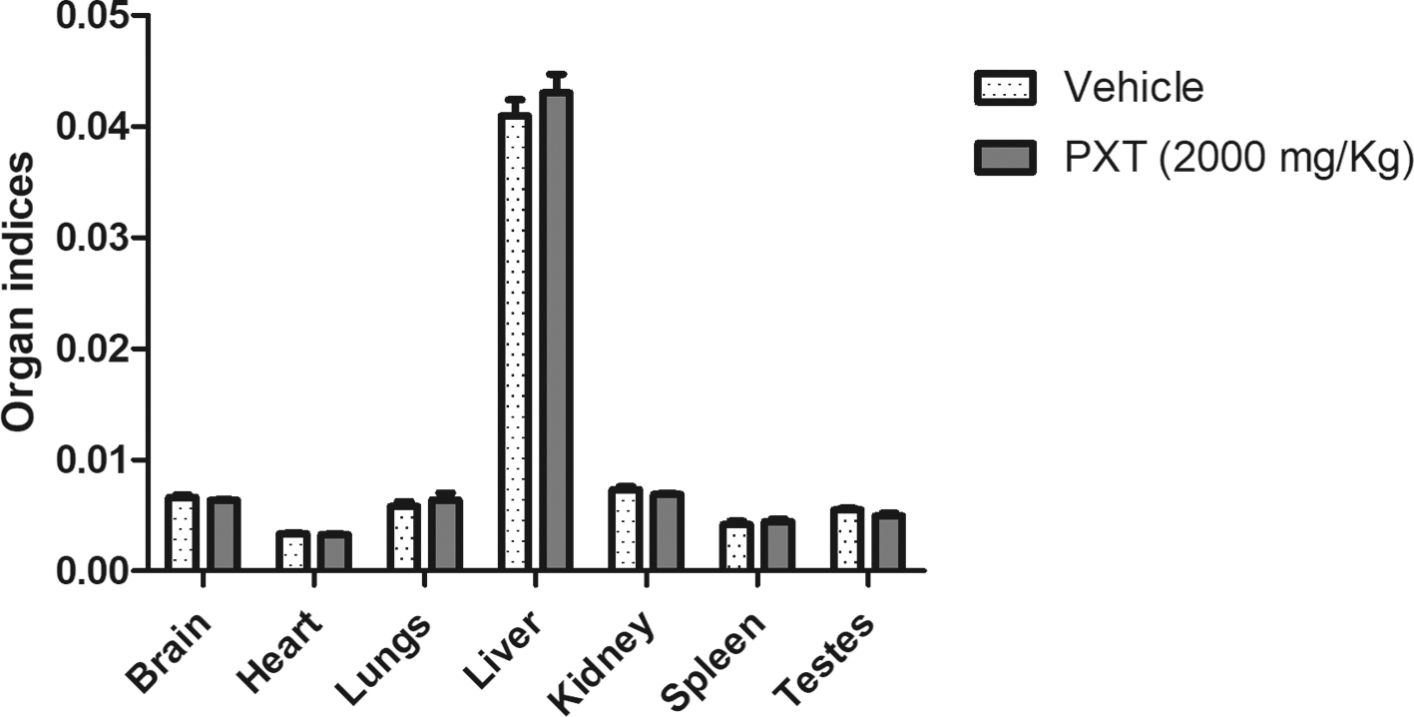
Acute oral toxicity analysis for PXT. The relative organ weights (organ indices) of male rats treated with a single dose of PXT for 14 days. Data represented as mean ± SEM (n=3). One-way ANOVA followed by Tukey’s multiple comparison test. No significant difference observed among the groups.

The results of the acute toxicity test revealed that treatment with PXT did not show any changes in behavioral patterns, skin, eyes, lacrimation, grooming signs, or postural reflex. No significant changes in body weight were observed during the follow-up period of 14 days. Importantly, all rats survived and no adverse effects were observed. Furthermore, no significant differences were observed among the organ indices of the brain, heart, lungs, liver, kidney, spleen, and testes between the vehicle- and PXT-treated groups ( Figure 1). Therefore, the limit test results suggest LD50 (lethal dose for 50% of the population) for the PXT formulation to be higher than 2000 mg/kg.

### 3.2 Sexual behavior

**Figure 2.**
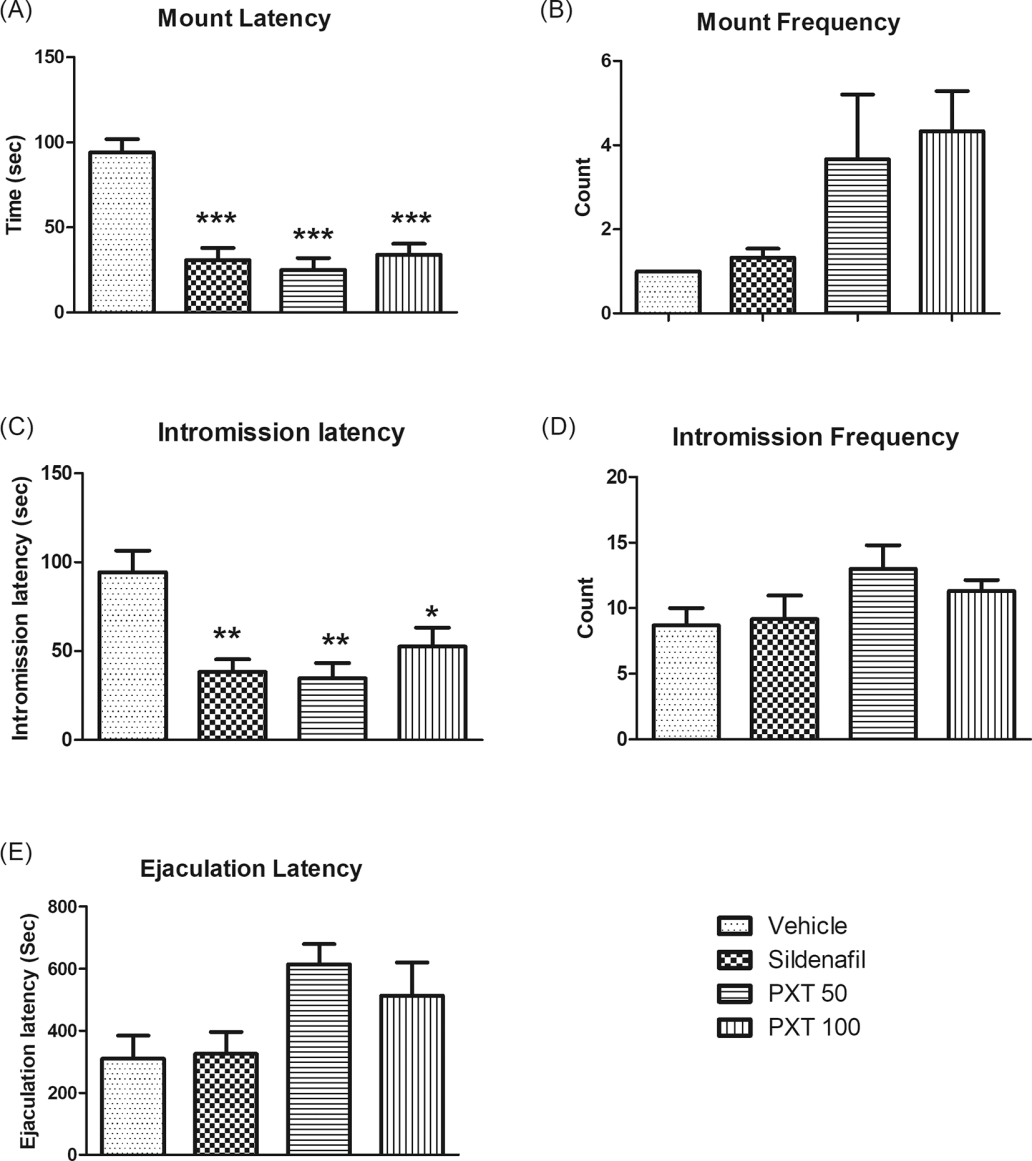
Effect of PXT treatment over sexual behavior in male rats. Data represented as mean ± SEM (n=6). One-way ANOVA followed by Tukey’s multiple comparison test. *p<0.05, **p<0.01 and ***p<0.001 as compared with control.

The effect of PXT on sexual activity was assessed by evaluating the copulatory behavior of experienced male rats. All the treatment groups, PXT 50 mg/kg, PXT 100 mg/kg, and sildenafil, exhibited a significant decrease in the latency for mounting (p<0.0001) and intromission (adjusted p = 0.0032, 0.0432, 0.0055, respectively) compared to the control group ( Figure 2A, C). Furthermore, a marked increase (non-significant) in mount frequency was observed in the PXT 50 and PXT 100 treatment groups compared to that in the vehicle and sildenafil groups ( Figure 2B). However, no significant difference in intromission frequency was observed between groups ( Figure 2D). The PXT 50 and PXT 100 groups exhibited increased ejaculation latency compared with the control and sildenafil groups ( Figure 2E). These results indicate that oral administration of 50 mg/kg and 100 mg/kg PXT for 28 days resulted in aphrodisiac activity, as indicated by enhanced sexual desire, sexual arousal, and copulatory efficiency in normal rats.

### 3.3 Sperm motility

**Figure 3.**
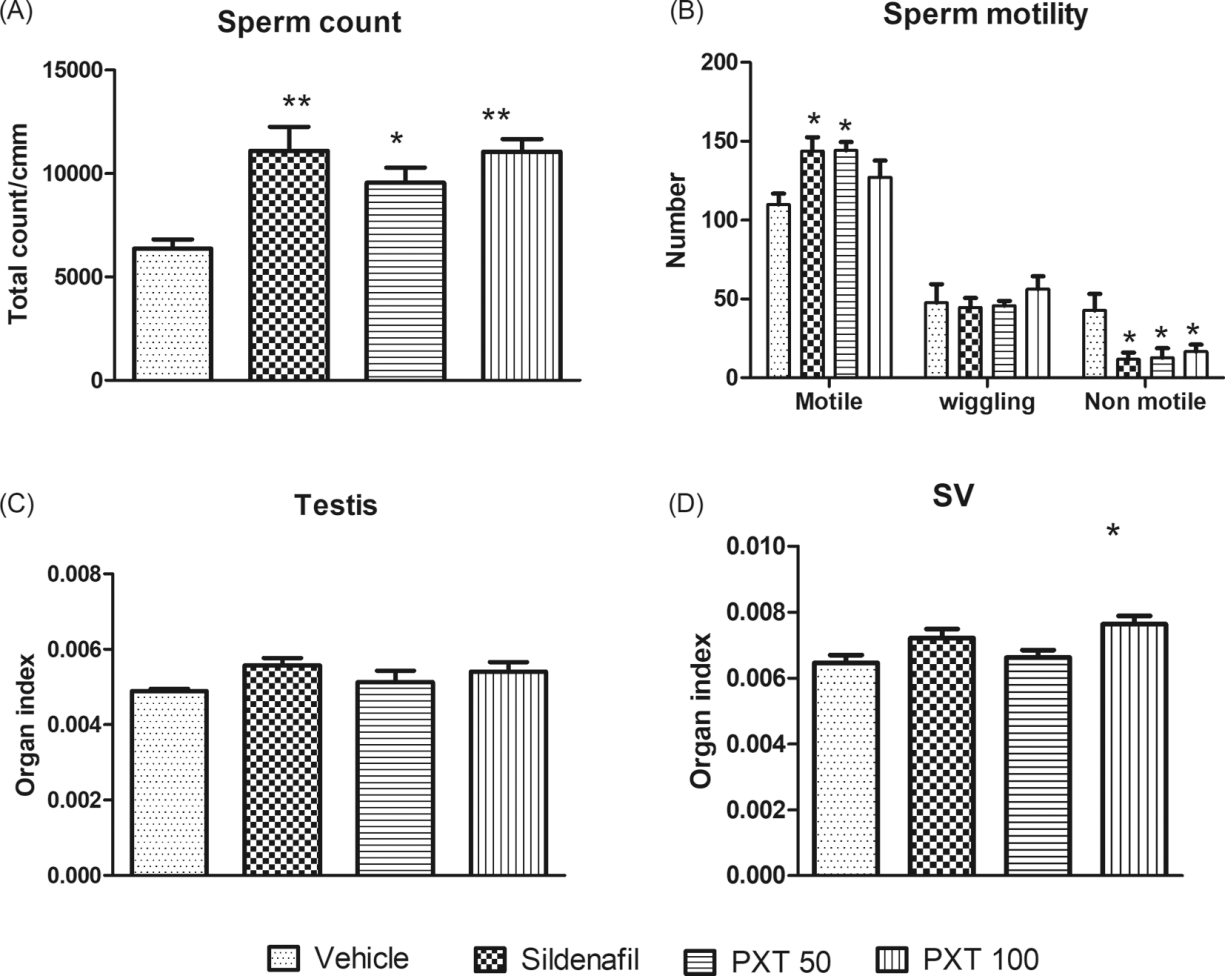
Effect of PXT treatment on reproductive parameters. (A) sperm count, (B) sperm motility, and (C&D) organ indices of testes and seminal vesicles (SV) in male rats. Data represent mean ± SEM (n=6). Data were analysed by one-way ANOVA followed by Tukey’s test. *p<0.05, **p<0.01 compared with control.

A semen analysis test was carried out to study the effect of PXT treatment on the quality and quantity of sperm (sperm number, morphology, and motility). Treatment groups, sildenafil and PXT 50 showed a significant increase (adjusted p=0.0072 and 0.0066, respectively) in the number of motile sperm compared to the control ( Figure 3B). Similarly, sildenafil, PXT 50, and PXT 100 significantly decreased (adjusted p=0.0150, 0.0200, and 0.0488, respectively) the number of non-motile sperm compared to the vehicle group ( Figure 3B). Additionally, all treatment groups, PXT 50 (adjusted p=0.0256), PXT 100 (adjusted p=0.0012), and sildenafil (adjusted p=0.0011), demonstrated a significant increase in the sperm population compared to the vehicle group ( Figure 3A). These results indicated that both doses of PXT and sildenafil possess spermatogenic potential.

### 3.4 Percentage occurrence of individual sperm abnormalities

Various sperm abnormalities, such as headless tail, bent neck, kinked tail, and tailless head, were observed in all groups. Among these abnormalities, bent neck and kinked tails constituted the highest percentage. The vehicle and PXT 50 groups showed a lower percentage of sperm with abnormalities than the sildenafil and PXT 100 groups. Although not statistically significant, PXT 100 showed a higher percentage of sperm with abnormalities than the control and PXT 50 groups ( Table 2).

**Table 2.** Percentage of sperm abnormalities across various groups.

	Vehicle	Sildenafil	PXT 50	PXT 100
Head less tail	1.25 ± 0.73	1.58 ± 0.95	0.83 ± 0.42	1.42 ± 0.91
Bent neck	2.50 ± 1.12	3.67 ± 1.40	2.42 ± 1.10	4.67 ± 1.39
Kinked tail	2.00 ±1.10	3.92 ± 0.92	2.67 ± 1.21	4.08 ± 0.99
Tail less head	1.00 ± 0.68	1.75 ± 0.91	0.75 ± 0.48	2.17 ± 0.83

All the values represent mean ± SEM of six animals.

### 3.5 Organ indices of testes and seminal vesicles

To estimate reproductive activity, indices of the reproductive organs were assessed. Although no major differences in testicular weight were observed between the control and treatment groups, a significant increase (adjusted p=0.0167) in the organ index of seminal vesicles was observed in the PXT 100 group compared to the control group ( Figure 3C). This finding suggests that PXT treatment can play a role in improving seminal secretions that affect androgenic function.

### 3.6 Histology of testes

**Figure 4.**
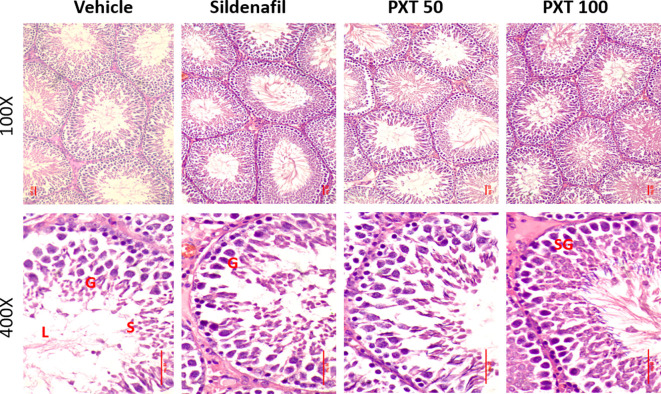
Representative photographs showing H&E stained testicular histology. Intact seminiferous epithelium can be appreciated in all the groups. No signs of degeneration or abnormalities were found. G represents germinal cells, S represents spermatids and sperm cells, L represents lumen and SG represents spermatogonial cells.

To assess the reproductive toxicity of PXT following chronic treatment, histological analysis of the testes was performed. Hematoxylin and eosin staining revealed that the tissue architecture and cellular integrity of the testes were maintained in the vehicle and treatment groups. The sections revealed numerous seminiferous tubules with several layers of cells, including Sertoli cells, spermatogonia, spermatocytes, and spermatids. Importantly, all levels of spermatogenic cells were orderly and closely arranged in rows, and no histological changes such as necrosed or degenerated tubules, depletion and degeneration of germ cells, and retention of spermatids were observed in any of the treatment groups ( Figure 4). These findings indicated that the doses of PXT used were not associated with any reproductive toxicity. ^41^

### 3.7 Brain DA and 5-HT estimations

**Figure 5.**
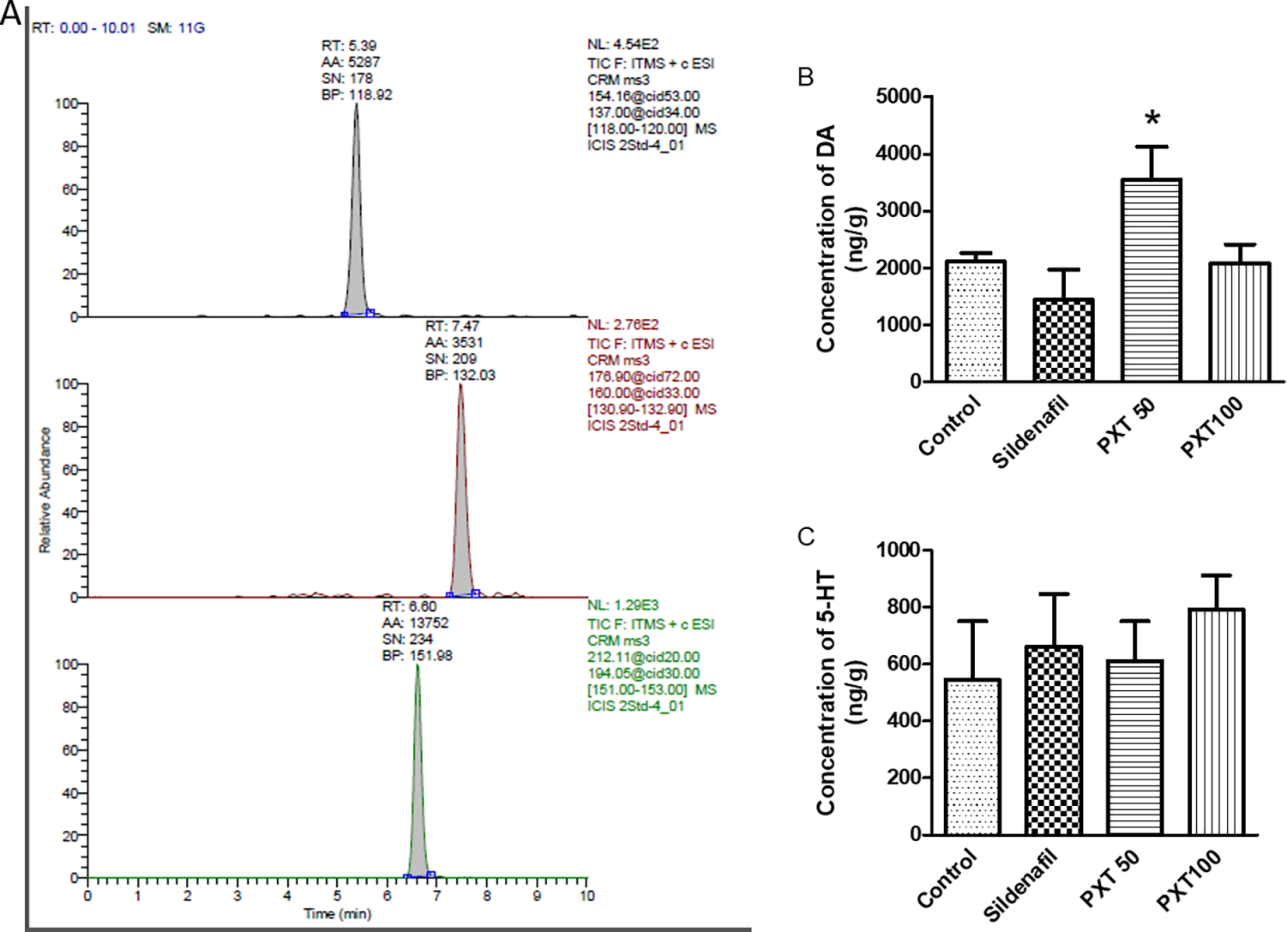
Effect of PXT treatment on dopamine and 5-HT levels. (A) Chromatogram of PXT treated rat brain sample. Retention time of dopamine (5.39 min), 5-HT (7.49 min) and isoprenaline (6.60 min). (B) Concentration of dopamine and (C) 5-HT in brain regions associated with sexual behavior assessed using LC-MS. Data represents mean ± SEM (n=4). Data analysed by one-way ANOVA followed by Tukey’s test. *p<0.05 compared with sildenafil.

The concentrations of serotonin and dopamine in the brain regions responsible for sexual activity were estimated. A representative chromatogram of the treated rat brain samples is shown in Figure 5A. In the MPOA and hypothalamus areas, DA concentrations were higher in the PXT50 group than in the control group (not significant). However, DA levels were significantly higher in the PXT50 group than in the sildenafil group (adjusted P=0.0340) ( Figure 5B). These results suggest that the administration of PXT (50 mg/kg) enhanced dopamine levels in the brain regions associated with sexual behavior, potentially leading to improved sexual activity. Conversely, no significant differences in serotonin levels were observed between the groups ( Figure 5C).

### 3.8 Bioactives and potential targets of PXT

A total of 126 chemical constituents of PXT were obtained from the USDA, IMPPAT, and a literature survey. Through ADME screening, 114 bioactive compounds with OB≥0.55 and more than two drug-likeness (Lipinski, Ghose, Veber, Egan, and Muegge) were chosen for further study. Furthermore, 916 unique targets were predicted for 114 bioactive compounds using STP, SEA, and PharmMapper.

### 3.9 Identification of bioactive targets involved in MSF

**Figure 6.**
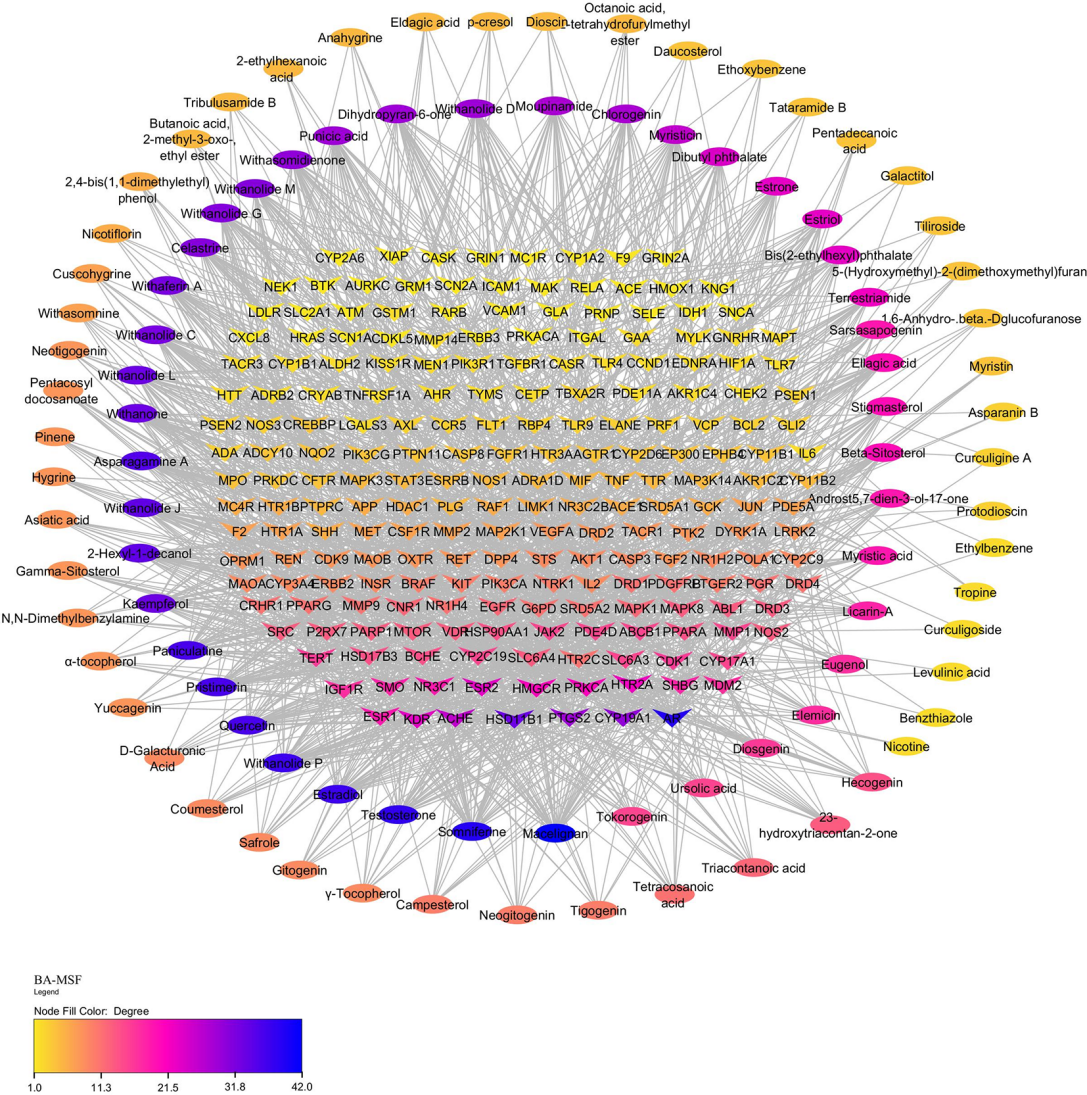
PXT-MSF common target network. Ellipses represent the potential bioactives of PXT that affect MSF. Arrow heads represent MSF-related target genes. Nodes are size sorted based on color.

Using “Male sexual function” as the search term, 1083 targets related to MSF were retrieved from the GeneCards, NCBI gene, and TTD databases. Among the 916 bioactive targets and 1007 MSF-related targets, 215 common targets were identified, indicating their potential involvement in MSF. The PXT-MSF common target network contained 327 nodes (94 bioactive, 223 target genes) and 1569 edges was established. Degree analysis indicated that macelignan, somniferine, estradiol, and testosterone possess a higher number of targets that could influence MSF. Furthermore, a significant number of bioactive compounds targeted key players such as the AR, CYP19A1 (aromatase), PTGS2 (cyclooxygenase), HSD11B1 (hydroxysteroid 11-beta dehydrogenase 1) and acetylcholinesterase (ACHE), suggesting their possible role in improving male sexual function ( Figure 6).

### 3.10 GO and KEGG enrichment analysis

**Figure 7.**
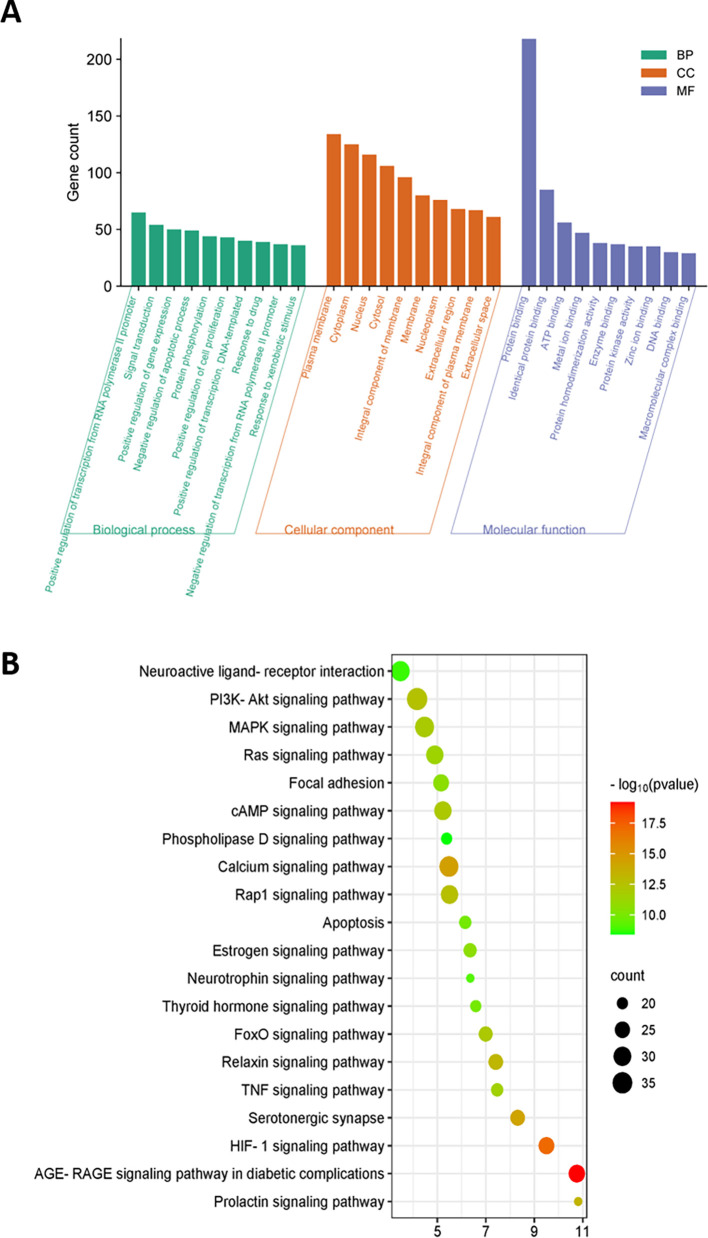
KEGG and GO enrichment analysis. (A) GO enrichment analysis of the target genes; (B) KEGG enrichment analysis of the target genes. The x-axis shows the number of genes enriched in the pathway. The size of bubbles represents the number of targets in the pathway and the color represents the p value.

GO enrichment analysis was performed to understand the biological information of 215 overlapping genes. This analysis revealed 832 enriched GO terms with a significance level of p<0.01, consisting of 648 biological processes, 75 cellular components, and 109 molecular functions ( Figure 7A). The principal biological processes include positive regulation of transcription from the RNA polymerase II promoter, signal transduction, and positive regulation of gene expression. Additionally, these genes were mainly related to functions such as protein binding, identical protein binding, and ATP binding. The major cellular components of these genes are the plasma membrane, cytoplasm, and the nucleus.

To explore the enriched signalling pathways related to the 215 candidate genes, KEGG pathway analysis was performed. The analysis identified that most targets were enriched in the PI3K-Akt signalling pathway, MAPK signalling pathway, and calcium signalling pathways, all of which are closely associated with male sexual function ( Figure 7B).

### 3.11 Construction of Compound-target-pathway (C-T-P) network

**Figure 8.**
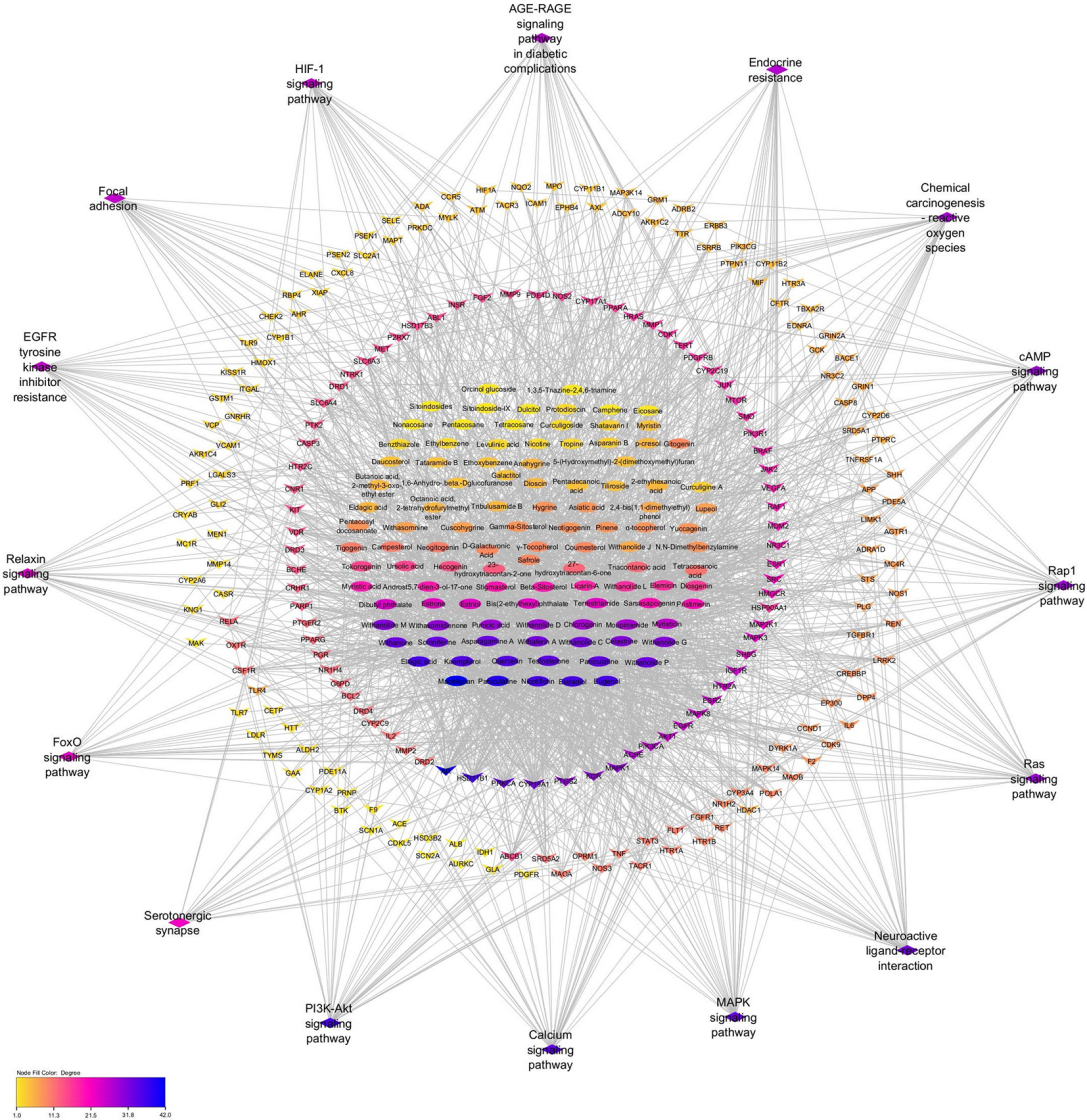
Compound-Target- pathway (C-T-P) network. Ellipses represent the potential bioactives of PXT that affect MSF. Diamonds represent the important pathways involved in PXT action toward MSF. Arrow heads represent MSF-related target genes. Nodes are size sorted by color.

The C-T-P network was built to provide a holistic understanding of PXT bioactives, their targets, and their associated pathways in male sexual function. As shown in Figure 8, the network was subjected to topological analysis. Based on the degree value, macelignan, paniculatine, nicotiflorin, estradiol, ellagic acid, testosterone, kaempferol, quercetin, paniculatine, and withanolide P were identified as 10 significant bioactive compounds with high degrees of MSF targets. AR, HSD11B1, PRKCA, CYP19A1, PTGS2, KDR, MAPK1, ACHE, and PIK3CA were identified as the top 10 crucial targets in the compound-target-pathway network ( Figure 8).

### 3.12 PPI network construction and core target screening

**Figure 9.**
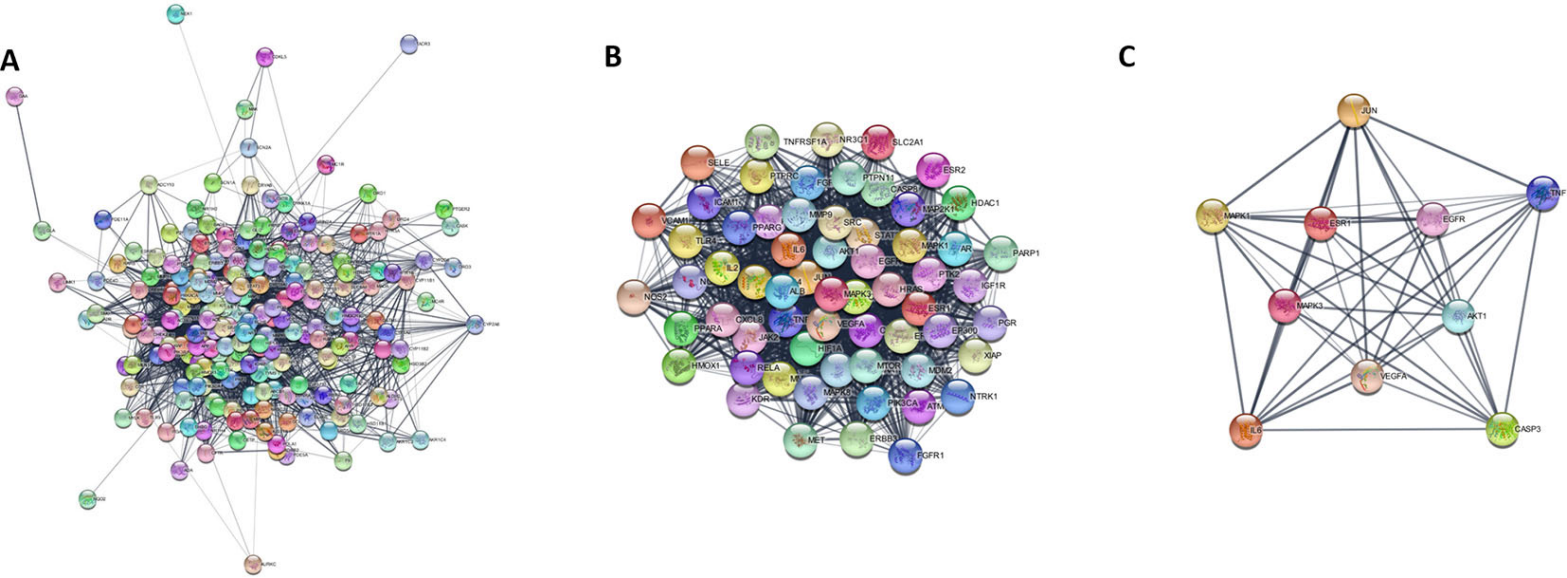
PPI subnetwork of PXT target genes for MSF. (A) PPI network of PXT target genes for MSF (B) Cluster with high cluster co-efficient generated after cluster analysis of PPI network; (C) Core targets obtained after ranking by MCC algorithm of Cytohubba application.

PPI network analysis was performed to identify key targets responsible for mediating the effects of PXT on MSF. The 215 candidate genes involved in MSF were used to construct a PPI with a confidence (score) cut-off of 0.4. A PPI interaction network consisting of 215 nodes and 3827 edges was generated in the cytosol ( Figure 9A). MCODE cluster analysis of the PPI network resulted in 11 clusters. Cluster 1 had the highest clustering coefficient of 45.279 and was therefore considered as the prominent subnetwork of the PPI ( Figure 9B). The core targets of the cluster 1 subnetwork were analyzed using the MCC′ algorithm of the Cytohubba plug-in. Analysis revealed AKT1, MAPK3, EGFR, ESR1, and VEGF as core targets involved in the action of PXT against MSF ( Figure 9C). In addition, these targets are associated with the PI3K-AKT pathway, which is the top signalling pathway involved in PXT action. Because AR is the most significant target in the PXT-MSF and C-T-P networks, it is also included as the key target.

### 3.13 Molecular docking

**Figure 10.**
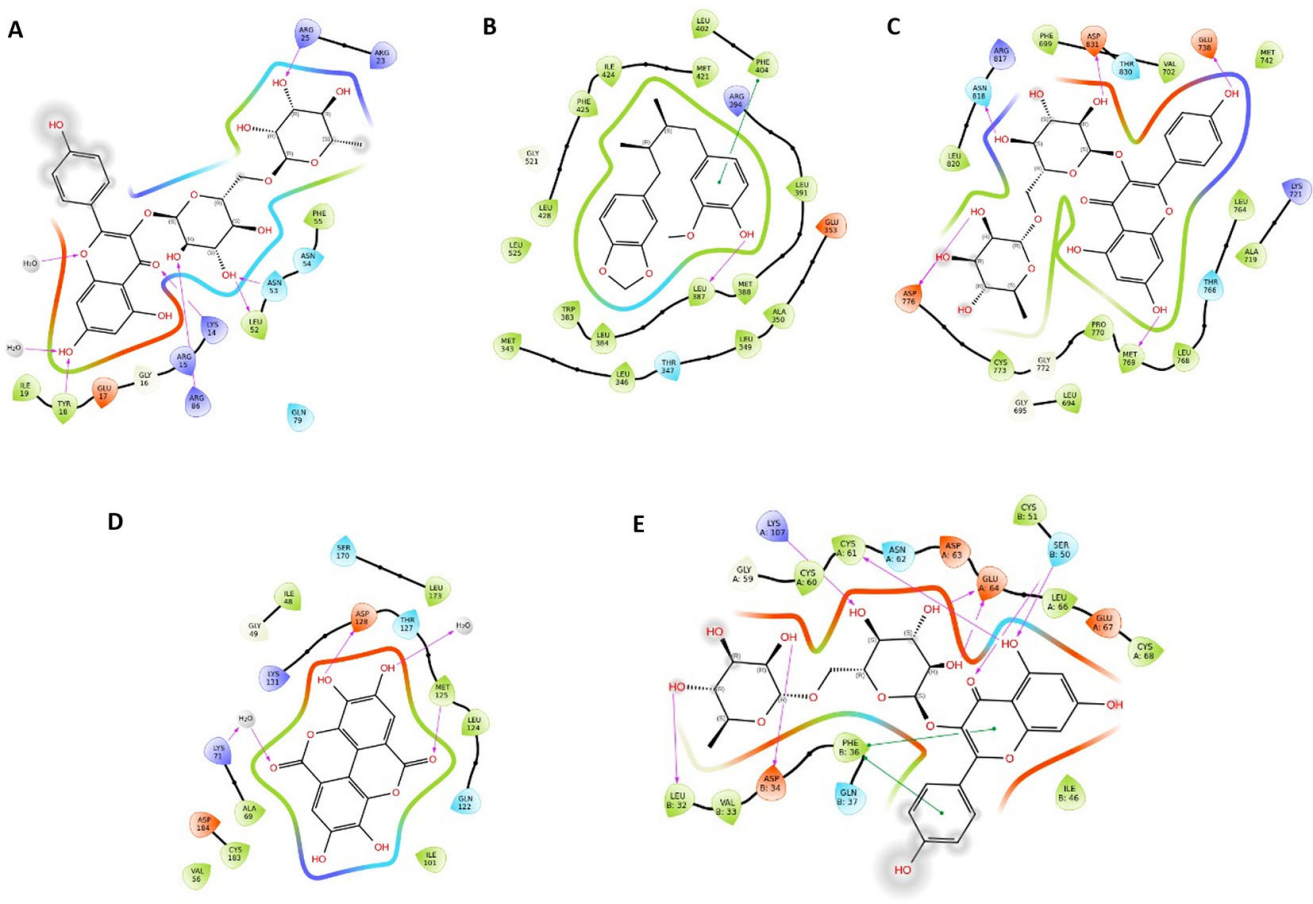
2D interaction diagram showing the types of contacts between core targets and the key bioactives. (A) Nicotiflorine-AKT1; (B) Ellagic acid-ESR1; (C) Nicotiflorine-EGFR; (D) Ellagic acid-MAPK3; (E) Nicotiflorine-VEGF. Purple arrows indicate hydrogen bonding. Green lines indicate pi-pi interactions.

Following the network analysis results, molecular docking analysis was conducted to validate the binding between the core targets (AKT1, VEGFA, MAPK3, ESR1, AR, and EGFR) and key bioactive compounds obtained from the C-T-P network. Table 3 displays the binding scores, which indicate the potential binding of most bioactive compounds to the active sites of their respective targets. Figure 10 illustrates the molecular interactions of bioactive compounds with the highest scores. The amino acid interactions of proteins with their respective ligands and their hydrogen bond lengths are listed ( Table 3). Most bioactive compounds exhibited hydrophobic and polar interactions with core targets. Quercetin docked with AKT1 through hydrogen bonding with inhibitory A. A residues, ARG86, and ASN53. In addition, the docking score and binding affinity were better than those of the known Akt1 inhibitor capivasertib. Testosterone, followed by beta-estradiol and quercetin, exhibited better binding affinity and docking score with AR, forming hydrogen bonds with the A. A residues ASN705, THR877, and GLN711 required for activation. Ellagic acid showed a better binding affinity with ESR1. Nicotiflorine, followed by quercetin and kaempferol, showed better binding affinities for EGFR and VEGF. Quercetin and ellagic acid showed improved binding to MAPK3.

**Table 3.** Binding scores of bioactives and core targets.

Protein (PDB)	Ligand	Docking score	Binding affinity	Interacting amino acids
**AKT1** (2UVM)	Nicotiflorine	-7.618	-14.89	2.6(ARG86), 2.13(LEU52), 2.17(ASN53),2.7(ARG 25), 2.25(TYR18), 2.03 (LYS14)
	Quercetin	-5.667	-28.05	2.25(ARG86), 2.15(ASN53),1.69 (ARG23),1.95(TYR18), 2.11and 2.55(LYS14)
	Capivasertib *	-5.341	-25.62	1.76(ARG86), 1.87(ASN53), 2.2 (LEU52)
**AR** (1T65)	Beta estradiol	-11.758	-77.35	1.74(ASN705), 1.86(THR877), 4.98Pi-Pi (PHE764)
	Testosterone *	-11.453	-83.05	1.93(ASN705), 2.24(THR877),2.38(GLN711), 2.6(ARG752)
	Ellagic acid	-11.357	-46.14	1.99(ASN705), 2.15(THR877)
	Quercetin	-11.067	-55.96	2.5(ASN705), 1.73(GLN711), 5.29Pi-Pi (PHE764)
	Macelignan	-10.439	-52.77	2.03(GLN711)
	Kaempferol	-8.835	-46.13	2.14(ASN705), 5.32Pi-Pi (PHE764)
	Paniculatine	-8.330	-44.17	Hydrophobic interactions
**ESR1** (2BJ4)	Beta estradiol *	-10.239	-68.67	2.23(LEU387), 5.36 Pi-Pi (PHE404)
	Ellagic acid	-10.230	-50.75	2.15(GLU353), 1.81(LEU346), 5.45 Pi-Pi (PHE404)
	Macelignan	-9.786	-46.14	2.79(GLU353), 5.15 Pi-Pi (PHE 404)
	Testosterone	-9.313	-56.92	No interactions
	Quercetin	-8.801	-32.74	1.84(ASP351), 1.97(THR347)
	Paniculatine	-8.468	-52.00	No interactions
	Nicotiflorine	-7.709	-21.36	1.59(ASP351)
	Kaempferol	-7.443	-32.76	1.80(ASP351)
**EGFR** (1M17)	Afatinib *	-10.24	-52.53	2.77(MET769), 1.55(ASP776), Salt bridge4.39(ASP776), 2.05(CYS773), Halogen bond3.38 (LEU764)
	Nicotiflorine	-10.197	-35.76	1.65(MET769), 2.13(ASP776), 2.65(ASP831), 1.64(GLU738), 2.39(ASN818)
	Quercetin	-9.902	-36.07	1.91 and 1.60(MET769), 1.70 and 1.08(ASP831)
	Kaempferol	-8.165	-41.04	1.96 and 1.23(MET769), 2.32(GLU738)
	Ellagic acid	-7.574	-41.82	1.88(MET769), 1.95 and 1.84(ASP831)
	Macelignan	-7.181	-38.29	2.11(MET769), 2.52(GLU738)
	Beta estradiol	-5.969	-29.2	1.92 and 2.06(MET769)
	Testosterone	-5.318	-30.98	2.12(MET769)
**MAPK3** (2ZOQ)	PD98059 *	-5.44	-52.32	2.39(ASP184), 2.68(MET125)
	Quercetin	-9.341	-34.86	1.8(ASP84), 2.51.7 (MET125)
	Ellagic acid	-9.137	-46.19	2.03(ASP128), 2.02(MET125)
	Kaempferol	-8.057	-34.57	5.45 Pi -Cat (LYS131), 2.53(ASP123)
**VEGF** (1VPF)	Pazopanib *	-3.511	-41.38	2.03(GLYA:59, 2.44(CYSA:68), 2.12(ASPB:34)
	Nicotiflorine	-9.878	-32.22	2.26(LYSA:107),1.75(CYS61), 1.86(GLUA:64), 2.32(LeuB32), 1.76(ASPB34), 1.59(SERB:50), 2.12(SERB:50), 5.42(Pi-Pi PHE:36), 5.28(Pi-Pi PHE36)
	Quercetin	-6.709	-35.54	1.88(LEUA:66), 2.09(ASPB:34), 1.75
	Kaempferol	-6.148	-32.41	1.88(LEUA:66), 2.49(ASPB34)
	Ellagic acid	-5.628	-39.97	2.12(CYS61), 2.63(LYSA107)

* Represents known activator/inhibitor.

## 4. Discussion

Chronic exposure to diverse insults, such as lifestyle factors, occupational stressors, aging, and environmental toxins can gradually disrupt male sexual function. ^1^ Consequently, the development of a single drug to improve male sexual function poses a challenge. Moreover, the continuous consumption of such drugs may lead to numerous side effects. In contrast, traditional plant-based medicine, with its unique polypharmacology approach, offers significant advantages in treating complex and multifactorial diseases, such as sexual dysfunction. ^2^^,^^3^ In addition, it is a safer alternative that can be routinely consumed by a healthier population. PXT is an Ayurvedic formulation that enhances male sexual function in a healthy population. However, the principal bioactivity of PXT and the underlying mechanisms responsible for its aphrodisiac action remain unknown.

The current study demonstrated that oral administration of the PXT formulation is safe up to 2000 mg/kg. Administration of PXT (50 mg/kg and 100 mg/kg) to male Wistar rats enhanced libido (sexual desire) and sexual arousal, as evidenced by decreased mount and intromission latencies and increased mount and intromission frequency. Compared to the control, PXT-treated rats showed increased ejaculation latency and prolonged duration of coitus, which indicates an increase in sexual motivation and copulatory performance. Studies suggest that ejaculation latency below 300 s indicates premature ejaculation, and above 1200 s indicates delayed ejaculation in rats. ^42^ In the present study, PXT improved the ejaculation time of the rats within this range.

With respect to their integral role in various phases of sexual behavior, DA and 5-HT levels were estimated in the brain regions (MPOA and hypothalamus) associated with sexual behavior. DA serves as the principal neurotransmitter responsible for sexual motivation, copulatory efficiency, and genital reflexes, ^43^ whereas 5-HT is known to have an inhibitory role in sexual behavior. ^3^ Interestingly, PXT 50 mg/kg PXT increased brain DA concentrations without altering 5-HT levels. This indicates that PXT 50 facilitates copulatory behavior by enhancing the dopamine tone. Similarly, a previous study reported that *E. longifolia* treatment elevated cortical and hippocampal dopamine levels, whereas 5-HT levels remained unaffected. ^44^

Notably, the effect of PXT 50 on mount latency, intromission latency, and intromission frequency was slightly more pronounced than with PXT 100. This difference might be attributed to a relatively greater increase in brain dopamine levels induced by PXT 50 compared to PXT 100. While the reduced dopamine levels by PXT 100 could be as a result of enhanced dopamine metabolism, possibly due to compensatory mechanisms that are activated at higher doses.

Furthermore, 50 mg/kg and 100 mg/kg PXT exhibited spermatogenic potential with increased viable sperm count and motility, which could be advantageous in treating oligospermia. However, an increased percentage of sperm abnormalities was observed in the 100 mg/kg PXT group compared to that in the other groups. Nonetheless, this was below 10%, indicating that PXT is free of reproductive toxicity at the recommended doses, even after treatment for 56 days. This was also supported by the preserved normal histology of the testes in PXT-treated rats.

Although several studies have indicated the aphrodisiac efficacy of whole plant extracts, limited knowledge exists regarding the exact bioactive compounds responsible for its beneficial action. To explore the bioactives, mechanisms, and molecular targets underlying the beneficial effects of PXT on male sexual function, we employed an integrated network pharmacology and molecular docking approach. The compound-target-pathway network analysis revealed that macelignan, paniculatine, nicotiflorin, β-estradiol, ellagic acid, testosterone, kaempferol, quercetin, paniculatine, and withanolide P had the maximum number of targets that could influence male sexual function. Previous studies have demonstrated that a few plant compounds, including beta-estradiol, ^45^ ellagic acid, ^46^ and kaempferol, ^47^ improve fertility and male sexual function in animal models. Quercetin has also been shown to improve male sexual function in several animal models. ^48^ However, the mechanisms by which they exert their actions are limited.

Our study demonstrated that PI3K-Akt signalling and MAPK signalling pathways might be crucial among the multiple signalling pathways through which PXT may improve male sexual function. Supporting studies have indicated that male reproductive functions, including the endocrine function of gonads and spermatogenesis, are regulated by PI3K-Akt signalling, and its dysregulation has been implicated in erectile dysfunction. ^49^^,^^50^ Through PPI interactome analysis, AKT1, VEGFA, ESR1, and EGFR were found to be the key targets of PXT in MSF. AR was also considered as a key target because it had the highest degree in the C-T-P and PXT-MSF common target networks.

Akt is a serine/threonine protein kinase that plays a fundamental role in cellular processes such as proliferation, cell growth, metabolism, and apoptosis. The Akt1 isotype is involved in spermatogenesis and testicular development. ^51^^,^^52^ Studies have also suggested that PI3K/AKT1 signalling through eNOS activation is responsible for penile erection and male sexual activity. ^53^ Our findings showed that quercetin and nicotiflorine had good binding affinities with Akt1. Supporting studies have also suggested that quercetin improves testicular injury and reproductive performance by attenuating oxidative stress via AKT modulation. ^54^ This suggests that PXT might improve MSF through a PI3-kinase/Akt signalling mechanism.

In addition to PI3-kinase/Akt signalling, androgen and estrogen signalling via the AR and ESR are critical for male gonad development, erectile function, and spermatogenesis during development and adulthood. ^55^ In addition to estradiol and testosterone, which are endogenous ligands of AR and ESR1, respectively, the current study showed that many phytoconstituents of PXT, such as ellagic acid, macelignan, and kaempferol, exhibited good binding affinities with AR and ESR1. This suggests that PXT may improve MSF through androgen and estrogen signalling.

Furthermore, VEGF is reported to have a direct trophic effect on penile smooth muscles, and treatment with VEGF supplementation is administered for erectile dysfunction of smooth muscle integrity issues. ^56^ The current study showed a good binding affinity of ellagic acid, quercetin, and kaempferol to VEGF. Additionally, MAPK signalling has been linked to erectile dysfunction by promoting endothelial dysfunction, apoptosis, and fibrosis. ^57^ Quercetin and ellagic acid showed good binding affinities for MAPK3. This suggests that PXT might improve MSF through the VEGF and MAPK3 signalling pathways.

## 5. Conclusion

Altogether, our study results suggest that PXT could be a safer and promising alternative that exhibits aphrodisiac and spermatogenic potential. This could be partly attributed to its ability to enhance the dopaminergic tone. In this study, we identified macelignan, estradiol, testosterone, quercetin, and ellagic acid as potential bioactive compounds in PXT, acting through multiple mechanisms to enhance male sexual function.

## Declarations

### Ethics approval

This study was approved by the Institutional Animal Ethics Committee (IAEC/KMC/50/2022).

## Consent for publication

Not applicable.

## Data Availability

Figshare: Data related to Network pharmacology studies, https://doi.org/10.6084/m9.figshare.24711369.v1. ^19^ The data contains the following underlying data
• PXT compounds and targets: targets related to the PXT bioactives • Disease genes: Genes involved in Male sexual function • Network analysis: Common targets, protein-protein interactions, network analysis, identification of hubs, pathways through which PXT might modulate the male sexual function, docking scores Creative Commons Attribution 4.0 International license (CC-BY 4.0) Supplementary data: HPTLC fingerprints of each plant present in the PXT formulation Figshare: Effect on testicular histology, https://doi.org/10.6084/m9.figshare.25249192.v1. ^41^ The data contains the representative photographs showing H&E stained testicular histology. CC BY 4.0 Figshare: ARRIVE checklist for ‘Poweromin X Ten, a polyherbal formulation improves male sexual function: *in vivo* and network pharmacology study’ https://doi.org/10.6084/m9.figshare.24763458.v1 ^18^ Data are available under the terms of the Creative Commons Attribution 4.0 International license (CC-BY 4.0).
