# Supplementary material for: Poweromin X Ten, a polyherbal formulation improves male sexual function: In vivo and network pharmacology study
Source: F1000Res. 2024 Aug 28;13:260. Originally published 2024 Apr 12. [Version 2] doi: 10.12688/f1000research.145889.2 (PMC11362716; doi:10.12688/f1000research.145889.2)
Supplement: Peer review history

# Reviewer report: Reviewer response for version 2

**DOI:** 10.5256/f1000research.170813.r317864

**Published:** 2024-08-29

**Authors:** Akingbolabo Daniel Ogunlakin

**Affiliations:** 1 Bowen University, Osun state, Nigeria

All the comment have been resolved. Thank you.

Is the work clearly and accurately presented and does it cite the current literature?

Yes

If applicable, is the statistical analysis and its interpretation appropriate?

Yes

Are all the source data underlying the results available to ensure full reproducibility?

Yes

Is the study design appropriate and is the work technically sound?

Yes

Are the conclusions drawn adequately supported by the results?

Yes

Are sufficient details of methods and analysis provided to allow replication by others?

Yes

Reviewer Expertise:

Pharmacognosy, and Biochemistry

I confirm that I have read this submission and believe that I have an appropriate level of expertise to confirm that it is of an acceptable scientific standard.

# Reviewer report: Reviewer response for version 1

**DOI:** 10.5256/f1000research.159907.r293968

**Published:** 2024-08-20

**Authors:** Idris Adewale Ahmed

**Affiliations:** 1 Lincoln University College, Petaling Jaya, Malaysia

In the manuscript titled: “Poweromin X Ten, a polyherbal formulation improves male sexual function: In vivo and network pharmacology study”, the authors investigated the toxicity and aphrodisiac potential of PXT in male rats and explored its principal mechanisms of action.

 The general logic of the article is fairly integral, however, some issues require clarification, especially in the methodology and result sections.

- The title is appropriate and the abstract is comprehensive.
- The introduction and the methodology are concise and well-presented.
- The composition of the standard pellet, however, should be highlighted.
- There were some inconsistencies in the use of symbols: “l” or “L; “min” or “minutes”.
- The sub-section 2.7.1 uses “Capitalize each word” in contrast to the rest of the sections and sub-sections (sentence case).
- In 3.1, the authors stated that “no significant differences were observed among the organ indices of the brain, heart, lungs, liver, kidney, spleen, and testes between the vehicle- and PXT-treated groups (Figure 1).”, however, the increase in the liver and spleen should be highlighted and some explanations on the possible causes should be provided.
- In 3.2: for the intromission frequency and 3.7: for DA concentration; the 50 mg/kg group had higher results than the 100 mg/kg groups. Explanations should be provided for this.
- Some claims in the “Discussion” especially the first paragraph should be substantiated with references.

Is the work clearly and accurately presented and does it cite the current literature?

Yes

If applicable, is the statistical analysis and its interpretation appropriate?

Yes

Are all the source data underlying the results available to ensure full reproducibility?

Yes

Is the study design appropriate and is the work technically sound?

Yes

Are the conclusions drawn adequately supported by the results?

Yes

Are sufficient details of methods and analysis provided to allow replication by others?

Yes

Reviewer Expertise:

Pharmacology

I confirm that I have read this submission and believe that I have an appropriate level of expertise to confirm that it is of an acceptable scientific standard.

# Response

**Published:** 2024-08-21

**Authors:** Sree Bojja

**Affiliations:** Pharmacology, Manipal Academy of Higher Education, Manipal, Karnataka, India

**Comment1:  The composition of the standard pellet, however, should be highlighted.**

**Response:** PXT is an Ayurvedic Proprietary Medicine of Apex laboratories Pvt. Ltd, and therefore, the standardized composition cannot be disclosed in the manuscript

**Comment 2:** **There were some inconsistencies in the use of symbol: “l” or “L:; “min” or “minutes”.**

**Response:** The terms ‘minutes’ and ‘L’ are now used uniformly.

**Comment 3: The sub-section 2.7.1 uses “Capitalize each word” in contrast to the rest of sections and sub-sections (sentence case).**

 Response: 2.7.1 Title is now corrected.

**Comment 4:** **In 3.1, the authors stated that “no significant differences were observed among the organ indices of the brain, heart, lungs, liver, kidney, spleen, and testes between the vehicle- and PXT-treated groups (Figure 1).”, however, the increase in the liver and spleen should be highlighted and the some explanations on the possible causes should be provided.**

**Response:** We thank you for the attention. The observed increase in the liver and spleen organ indices compared to the control group was marginal and statistically insignificant. Therefore, it does not raise any significant concerns. However, this slight increase may indicate that the liver plays a major role in the metabolism of PXT.

**Comment 5: In 3.2: for the intromission frequency and 3.7: for DA concentration; the 50 mg/kg group had higher results than 100 mg/kg groups. Explanations should be provided for this, too.**

**Response:** Thank you for raising an important point. The following explanation is now added in the manuscript.

 ‘The effect of PXT 50mg/kg on mount latency, intromission latency, and intromission frequency was slightly more pronounced than with PXT 100mg/kg. This difference might be attributed to a relatively greater increase in brain dopamine levels induced by PXT 50mg/kg compared to PXT 100mg/kg. While the reduced dopamine levels by PXT 100mg/kg could be as a result of enhanced dopamine metabolism, possibly due to compensatory mechanisms that are activated at higher dosages.’

**Comment 6: Some claims in the “Discussion” especially in the first paragraph should be substantiated with relevant references.**

**Response:** References are added to the necessary statements in the discussion part.

# Reviewer report: Reviewer response for version 1

**DOI:** 10.5256/f1000research.159907.r271367

**Published:** 2024-07-17

**Authors:** Akingbolabo Daniel Ogunlakin

**Affiliations:** 1 Bowen University, Osun state, Nigeria

1.  Authors need to mention the determination of LD50 in the abstract.

 2. Preparation of standard extract of PXT

 Hydroalcoholic dry powder extracts processed from *W. somnifera root, A. racemosus root, P. granatum seed, M. fragrans mace, C. arundinaceum root, O. mascula root, C. orchoides rhizome, T. terrestris fruit, C. paniculatus seed*, and Shilajit exudate were obtained from Amsar Private Limited, India, and the details of their individual specifications and quality standards are provided in the repository. ^19^ The powders along with pharmaceutical excipients were combined at specified ratios into a filing-coated tablet of 800 ± 25 mg for Code No. HTB020, which was used.

 I wonder how the authors got the formation right since PXT is a formulation in the market. Is PXT their patent? This need to be clarified.

Is the work clearly and accurately presented and does it cite the current literature?

Yes

If applicable, is the statistical analysis and its interpretation appropriate?

Yes

Are all the source data underlying the results available to ensure full reproducibility?

Yes

Is the study design appropriate and is the work technically sound?

Yes

Are the conclusions drawn adequately supported by the results?

Yes

Are sufficient details of methods and analysis provided to allow replication by others?

Yes

Reviewer Expertise:

Pharmacognosy, and Biochemistry

I confirm that I have read this submission and believe that I have an appropriate level of expertise to confirm that it is of an acceptable scientific standard, however I have significant reservations, as outlined above.

# Response

**Published:** 2024-08-20

**Authors:** Sree Bojja

**Affiliations:** Pharmacology, Manipal Academy of Higher Education, Manipal, Karnataka, India

**Reviewer comment 1.  Authors need to mention the determination of LD50 in the abstract.**

 Response:  LD50 evaluation and results are now added to the abstract. ‘Acute oral toxicity evaluation of PXT indicated LD50 greater than 2000 mg/kg.’

**Comment 2. Preparation of standard extract of PXT. I wonder how the authors got the formation right since PXT is a formulation in the market. Is PXT their patent? This need to be clarified.**

 Response: PXT is an Ayurvedic Proprietary Medicine of Apex laboratories Pvt. Ltd, and the company has provided the composition of the formulation and its characterisation profile.
